# Prevalence and forms of aggressive behavior among patients admitted to an acute psychiatric ward

**DOI:** 10.1192/j.eurpsy.2022.1503

**Published:** 2022-09-01

**Authors:** H. Girasek, A. Soos, G. Gazdag

**Affiliations:** Jahn Ferenc South-pest Hospital, Centre Of Psychiatry, Budapest, Hungary

**Keywords:** aggressive behavior, Mental Disorders, risk assessment, acute psychiatric ward

## Abstract

**Introduction:**

Aggressive behavior is frequently associated with acute psychiatric admission. Several studies highlight the increased risk of aggression in certain psychiatric disorders.

**Objectives:**

The aim of the study was to explore the extent of aggressive behavior, its various manifestations, and its association with gender, age, and diagnosis among patients admitted to an acute psychiatric ward.

**Methods:**

Patients admitted to our acute psychiatric ward in a three months period were included and the Dynamic Appraisal of Situational Aggression - Inpatient Version (DASA-IV) questionnaire was administered in the first seven days after admission for all patients.

**Results:**

A total of 290 patients, 153 men and 137 women, with a mean age of 46.9 years (SD=17.5) participated in the study. Men were overrepresented among patients who showed aggressive behavior (p=.008). There was no correlation between age and DASA-IV score (p=.259). 40% of patients (N=116) did not show aggression, while 60% (N=174) experienced some form of aggression. Of those who exhibited some form of aggressive behavior, 94% had only low, 4% had high, and 2% had extremely high levels of aggression. Aggression was most common in patients with intellectual disabilities, dementia, and bipolar disorder.

**Conclusions:**

According to our findings the majority of the acutely admitted psychiatric patients shows no or only low level of aggression. There were also differences in the forms and extent of aggressive behavior between the diagnostic groups. Risk assessment is important because it provides an opportunity for early detection and prevention, and the development of personalized treatment plans.

**Disclosure:**

No significant relationships.

